# Antibacterial Secondary Metabolites from the Cave Sponge *Xestospongia* sp

**DOI:** 10.3390/md10051037

**Published:** 2012-05-07

**Authors:** Sridevi Ankisetty, Marc Slattery

**Affiliations:** Department of Pharmacognosy, School of Pharmacy, The University of Mississippi, University, MS 38677, USA; Email: ankisett@olemiss.edu (S.A.); slattery@olemiss.edu (M.S.); Tel.: +1-662-915-1706 (S.A.); +1-662-915-1053 (M.S.); Fax: +1-662-915-6975 (S.A.)

**Keywords:** *Xestospongia*, polyacetylenes, antibacterial activity

## Abstract

Chemical investigation of the cave sponge *Xestospongia* sp. resulted in the isolation of three new polyacetylenic long chain compounds along with two known metabolites. The structures of the new metabolites were established by NMR and MS analyses. The antibacterial activity of the new metabolites was also evaluated.

## 1. Introduction

Sponges of the genera *Haliclona*, *Adocia*, *Xestospongia*, *Strongylophora*, *Petrosia* belonging to the order Haplosclerida have been rich sources of long-chain polyacetylenic alcohols which can be considered as chemotaxonomic markers [1] and these represent a rapidly growing class of sponge metabolites. Common hydroxylated polyacetylenes and brominated C_18_ acetylenic acids are the major class of metabolites reported from the above genera. Polyacetylenes have been found to show a range of biological activities which include antimicrobial, cytotoxic, antitumor, antiviral, immunosuppressant, and enzyme inhibition [2,3,4,5,6,7,8,9,10,11,12,13]. Ecological significance of these compounds includes inducing metamorphosis of ascidian larvae, preventing fouling by barnacle larvae, or inhibiting fertilization of starfish gametes [14].

**Figure 1:**
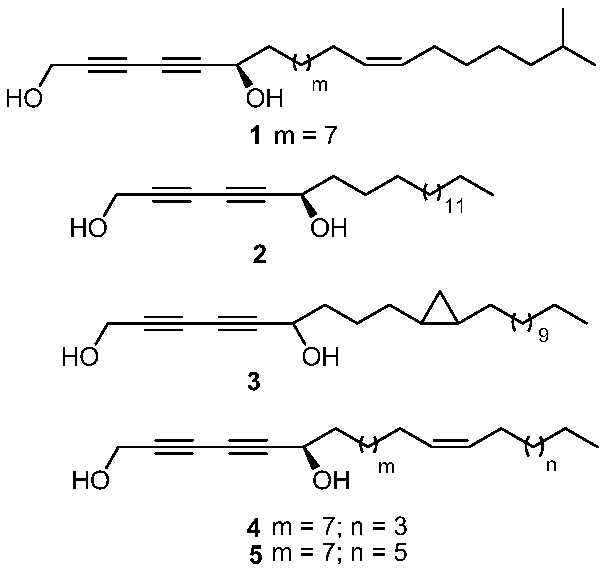
Structure of compounds **1**–**5**.

In the course of our search for bioactive metabolites from sponges, we have investigated the CH_2_Cl_2_/MeOH (1:1 v/v) extract of the cave sponge *Xestospongia* sp. collected from Pohnpei, Federated States of Micronesia, which exhibited antimicrobial activity against the gram-negative bacteria *Pseudomonas aeruginosa* and the gram-positive bacteria *Mycobacterium intracellulare* with IC_50_’s of 2.07 and 1.03 μg/mL, respectively. In this article we report the isolation and structure elucidation of three new metabolites (**1**–**3**) along with two known metabolites (**4**, **5**) (Figure 1) which belong to the class of polyacetylenic long chain compounds, as well as their antibacterial activities.

## 2. Results and Discussion

### 2.1. Bioassay-Guided Isolation

The crude DCM extract of the sponge which displayed antibacterial activity was subjected to reverse phase HPLC (Phenomenex, Luna C_18_(2)) using a gradient mixture (94:6 CH_3_CN:H_2_O to 100% CH_3_CN over 43 min) to afford two pure compounds (**4** and **5**). Rest of the peaks were further separated on a PhenylHexyl column (Phenomenex, Luna, 250 × 10 mm, 5 μm) using an isocratic elution system (80:20 CH_3_CN:H_2_O) to yield compounds (**1**–**3**).

### 2.2. Structural Elucidation of the New Compounds

Compound **1**, which was isolated as a colorless solid, had a molecular formula of C_24_H_40_O_2_, deduced by HRESIMS at *m/z* 383.2919 [M + Na]^+^ indicating five degrees of unsaturation. The IR spectra showed absorptions at 3292 and 2150 cm^−1^ which indicated the presence of hydroxyl and acetylene groups. The UV absorptions at 230, 241 and 253 nm indicated the presence of conjugated triple bonds. The ^13^C NMR and DEPT experiments revealed the presence of 24 carbon atoms. The ^1^H and ^13^C NMR spectra (Table 1) revealed signals due to a primary hydroxy group δ_H_ 4.37 (2H, s), δ_C_ 51.5 (CH_2_), a secondary hydroxy group δ_H_ 4.45 (1H, t), δ_C_ 62.9 (CH), four sp carbons, 69.0 (C), 69.6 (C), 77.5 (C), 80.7 (C), two sp^2^ carbons 129.9 (CH), 129.8 (CH), 5.37 (2H, t) and an isopropyl moiety. The position of the double bond was determined by examining the EIMS fragmentation pattern which indicated the presence of ion at *m/z* 257 [M − H_2_O − C_6_H_13_]^+^ which arose from the allylic cleavage of the side chain. The spectral data indicated similarities to the previously reported strongylodiol C [15] except that compound **1** had molecular formula less by two methylene units. Thus the structure of the new compound was established as **1**.

**Table 1:** Selective ^13^C NMR and ^1^H NMR data of compounds (**1**–**3**) ^a^.

1			2			3		
C#	δ_C_ mult	δ_H_ (*J* in Hz)	C#	δ_C_ mult	δ_H_ (*J* in Hz)	C#	δ_C_ mult	δ_H_ (*J* in Hz)
1	51.5 t	4.37 (s)	1	51.5 t	4.34 (s)	1	51.5 t	4.33 s
2	77.5 s	-	2	77.5 s	-	2	77.3 s	-
3	69.6 s	-	3	69.9 s	-	3	69.9 s	-
4	69.0 s	-	4	68.8 s	-	4	68.7 s	-
5	80.7 s	-	5	80.6 s	-	5	80.7 s	-
6	62.9 d	4.45 (t, 6.6)	6	62.9 d	4.43 (t, 6.4)	6	62.9 d	4.4 (t, 6.8)
7	37.7 t	1.74 m	7	37.8 t	1.75 m	7	37.5 t	1.7 m
16	129.9 d	5.37 (t, 4.5)	22	14.3 q	0.88 t (6.8)	10,11	15.8 d	0.63 m (2H)
17	129.8 d	5.37 (t, 4.5)	23	14.2 q	0.87 t (6.8)			
22	28.3 d	1.46 m	24	10.8 t	−0.35, 0.55 m			
23	22.6 q	0.89 d (6.5)						
24	22.6 q	0.89 d (6.5)						

^a^ data was run in CDCl_3_.

Compound **2** was obtained as colorless oil, had a molecular formula of C_22_H_38_O_2_ as indicated by HRESIMS at *m/z* 357.2761 [M + Na]^+^. This suggested four degrees of unsaturation which were satisfied by two acetylene groups. The structure of compound **2** was similar to that of **1** except for the absence of double bond, and the terminal isopropyl group being replaced by a terminal methyl group at δ_H_ 0.88 (3H, t), δ_C_ 14.3 (CH_3_). Thus the structure of the new compound was established as **2**.

Compound **3** was isolated as colorless solid, had a molecular formula of C_24_H_40_O_2_ as deduced by HRESIMS at *m/z* 383.2921 [M + Na]^+^ which indicated five degrees of unsaturation. In addition to the two triple bonds, one primary and one secondary hydroxy moieties three mutually coupled high field signals were observed in the ^1^H NMR spectrum [δ_H_ −0.35 (1H, m), 0.55 (1H, m), δ_C_ 10.8 and δ_H_ 0.63 (2H, m), δ_C_ 15.8] which were diagnostic for 1,2-disubstituted cyclopropane ring. The large difference in chemical shifts of the methylene group of the cyclopropane ring indicated *cis* stereochemistry of the three-membered cyclopropane ring which was confirmed by comparing ^1^H NMR values with reported data [16]. The position of the cyclopropane ring could not be determined as no HMBC correlations were seen from the cyclopropyl proton signals to the triple bond or to the long chain terminal methyl group which indicated that the cyclopropane moiety is present in the side chain not closer to any of the above mentioned moieties. Also due to very limited amounts of the isolated compound no chemical reactions could be carried out in order to determine the position of the cyclopropane ring. However this is the first report of isolation of cyclopropane containing long chain acetylenic alcohol.

Compounds **4** and **5** were identified as known compounds 18-hydroxyrenierin-2 [17] and strongylodiol A [15] by comparison of spectral data to the literature. Comparison of the specific rotation of compound **5** to that of the reported value indicated *R* configuration at C-6. By analogy the sign of specific rotation of all other compounds (**1**, **2** and **4**) also indicate *R*-configuration at C-6, which could be also true for compound **3** as it belongs to the same series of compounds. The isolated compounds were evaluated for antibacterial activity against *P. aeruginosa* and *M. intracellulare* and the results are presented in Table 2.

**Table 2:** Antibacterial assay data of pure compounds (**1**–**5**).

Compound	***P. aeruginosa*** **IC_50_**	***M. intracellulare*** IC_50_ *
1	1.7	9.9
2	1.9	7.7
3	1.8	14.3
4	2.6	23.0
5	2.9	17.5
Ciprofloxacin	0.2	1.5

* IC_50_ in μΜ.

## 3. Experimental Section

### 3.1. General

Optical rotations were measured using a JASCO DIP-370 digital polarimeter. UV spectra were recorded on a Hewlett-Packard 8452A diode array spectrometer. IR spectra were recorded on an ATI Mattson Genesis series FTIR spectrometer.NMR spectra were measured on Bruker Advance DRX-400 spectrometer. ^1^H and ^13^C NMR spectra were measured and reported in ppm using CDCl_3_ solvent peak (δ_H_ 7.24 and δ_C_ 77.23) as an internal standard. ESI-FTMS analyses were measured on a Bruker Magnex BioAPEX 30es ion cyclotron HR HPLC-FT spectrometer by direct injection into an electrospray interface. EIMS data was acquired on Waters VG 70–250S magnetic sector mass spectrometer. HPLC purifications were carried out on a Waters 2695 model system equipped with dual absorbance UV detector.

### 3.2. Sponge Material

The sponge (ID: PN10407137) was collected from a small cave at a depth of about 40 m. The samples exuded pigment and were a crème color. They were massive in shape with a “velvet-like” feel to their surface that was somewhat brittle upon collection. This new species is currently being identified by the Porifera Tree of Life (PorToL) project. Voucher specimen of the sample was deposited at the NOAA Ocean Biotechnology Center and Repository, Oxford, MS, USA.

### 3.3. Extraction and Isolation

43.0 g of freeze-dried and finely ground sponge material was exhaustively extracted with CH_2_Cl_2_/MeOH (1:1) to yield 4.4 g of the crude extract after concentration under reduced pressure. The crude extract was partitioned using dichloromethane (DCM) and water. The DCM fraction was dried to give 2.37 g of the extract. The crude DCM extract was subjected to repeated reverse phased semi-preparative HPLC purification on Phenomenex, Luna, C_18_(2), 10 μm, 250 × 21.2 mm; flow rate, 10 mL/min, using a gradient of 94:6 (CH_3_CN:H_2_O) to 100% CH_3_CN in 43 min. Peaks 3 and 8 were pure and identified as compounds **4** (2.0 mg) and **5** (3.5 mg). Peaks 4, 5 and 11 were further subjected to reverse phase HPLC purification on Phenomenex PhenylHexyl, 5 μm, 250 × 10 mm; flow rate, 3 mL/min; detector wavelength, 195 nm using isocratic conditions, 80:20 CH_3_CN:H_2_O to yield compounds **1** (1.5 mg), **2** (1.0 mg) and **3** (1.2 mg). 

Compound **1**: Colorless solid; [α]^25^_D_: −15.5 (*c* 0.11,CHCl_3_); UV (MeOH) λ_max_ (log ε) 230 (2.51) 241 (2.48) 253 nm (2.37); IR (NaCl) ν_max_ 3292, 2848, 2150, 1450, 1000 cm^−1^; ^1^H and ^13^C NMR data, see Table 1; EIMS [M]^+^/e 360 (1.5%), 342 [M − H_2_O]^+^; 2.1%, 257 [M − H_2_O − C_6_H_13_]^+^; 25%; HRESIMS *m/z* 383.2919 [M + Na]^+^ (calcd for C_24_H_40_O_2_Na^+^ 383.2926).

Compound **2**: Colorless oil; [α]^25^_D_: −17.8 (*c* 0.09,CHCl_3_); UV (MeOH) λ_max_ (log ε) 231 (2.47) 241 (2.38) 251 nm (2.29); IR (NaCl) ν_max_ 3194, 2859, 2198 cm^−1^; ^1^H and ^13^C NMR data, see Table 1; HRESIMS *m/z* 357.2761 [M + Na]^+^ (calcd for C_22_H_38_O_2_Na^+^ 357.2769).

Compound **3**: Colorless solid; [α]^25^_D_: −18.6 (*c* 0.05,CHCl_3_); UV (MeOH) λ_max_ (log ε) 230 (2.39) 243 nm (2.26); IR (NaCl) ν_max_ 3292, 2848, 2150, 1450, 1000 cm^−1^; ^1^H and ^13^C NMR data, see Table 1; HRESIMS *m/z* 383.2921 [M + Na]^+^ (calcd for C_24_H_40_O_2_Na^+^ 383.2926).

Compound **4**: [α]^25^_D_: −12.7 (*c* 0.12,CHCl_3_); HRESIMS *m/z* 369.2764 [M + Na]^+^ (calcd for C_23_H_38_O_2_Na^+^ 369.2769); ^1^H and ^13^C NMR data, see [16].

Compound **5**: [α]^25^_D_: −9.7 (*c* 0.11, CHCl_3_); [α]^25^_D_: −7.2 (*c* 1.11, CHCl_3_); HRESIMS *m/z* 397.3875 [M + Na]^+^ (calcd for C_25_H_42_O_2_Na^+^ 397.3082); ^1^H and ^13^C NMR data, see [15].

### 3.4. Antibacterial Assay

Organisms were obtained from the American Type Culture Collection (Manassas, VA, USA) which includes *Pseudomonas aeruginosa* ATCC 27853, and *Mycobacterium intracellulare* ATCC 23068. Susceptibility testing was performed using a modified version of the CLSI (formerly NCCLS) methods [18,19]. *M. intracellulare* was tested using a modified method of Franzblau *et al*. [20]. Samples were serially-diluted in 20% DMSO/saline and transferred in duplicate to 96 well flat bottom microplates. Microbial inocula were prepared by correcting the OD_630_ of microbe suspensions in incubation broth to afford final target inocula. Ciprofloxacin (ICN Biomedicals, Aurora, OH, USA) was included as the positive control. Organisms were read at 530 nm using the Biotek Powerwave XS plate reader (Bio-Tek Instruments, Winooski, VT, USA) (*P. aeruginosa*) or 544ex/590em, (*M. intracellulare*) using the Polarstar Galaxy Plate Reader (BMG Lab Technologies, Germany) prior to and after incubation. IC_50_’s (concentrations that afford 50% inhibition relative to controls) are calculated using XLfit 4.2 software (IDBS, Alameda, CA, USA, 2005) using fit model 201.

## 4. Conclusions

In summary, three new (**1**–**3**) and two known (**4**, **5**) metabolites were isolated from the sponge *Xestospongia* sp. and their structural elucidation was done using spectroscopic analysis. These metabolites exhibited moderate antibacterial activity against gram-negative bacteria *P. aeruginosa* and the gram-positive bacteria *M. intracellulare* when compared to the positive control.
